# Using AI in Forward-Backward Translation of Questionnaires for Men Invited to Prostate Cancer Screening: Methodological Study

**DOI:** 10.2196/81900

**Published:** 2026-02-26

**Authors:** Sofie Meyer Andersen, Pia Kirkegaard, Krzysztof Tupikowski, Katarzyna Hodyra-Stefaniak, Mette Bach Larsen, The PRAISE-U Consortium

**Affiliations:** 1 Department of Clinical Medicine Faculty of Health Aarhus University Aarhus N Denmark; 2 University Clinic for Cancer Screening, Department of Public Health Programmes Randers Regional Hospital Randers NOE Denmark; 3 Subdivision of Urology, Lower Silesian Center for Oncology, Pulmonology and Hematology Wrocław Poland; 4 Department of Oncology and Haematology Medical Faculty Wroclaw University of Science and Technology Wrocław Poland; 5 Research and Development Department, Lower Silesian Center for Oncology, Pulmonology and Hematology Wrocław Poland; 6 Department of Oncology, Lillebaelt Hospital Vejle Denmark; 7 See Acknowledgments

**Keywords:** artificial intelligence, AI, forward-backward translation, questionnaire adaptation, prostate cancer screening, cross-cultural research, cognitive interviews, health survey translation

## Abstract

**Background:**

Translation is important in research to ensure cultural relevance, accuracy, and generalizability, particularly in cross-cultural studies. The forward-backward translation method of the World Health Organization (WHO) is commonly used to improve linguistic and conceptual accuracy but is often time-consuming and resource intensive. The development of advanced artificial intelligence (AI) offers new opportunities to make the translation process more efficient, potentially reducing time and costs. However, concerns remain regarding the ability of AI to capture cultural nuances and complex linguistic structures, which may affect translation quality. Therefore, evidence on how AI can be effectively integrated into established translation frameworks remains limited.

**Objective:**

This study aimed to explore the use of AI in the forward-backward translation process for questionnaires.

**Methods:**

We used an adapted version of the WHO 4-step forward-backward translation method to translate the questionnaires from English into Polish. The questionnaires included the Prostate Cancer Screening Education (PROCASE) Knowledge Index, the Attitude Scale, Risk Perception items, and the Brief Health Literacy Scale for Adults. First, 2 AI tools (ChatGPT [GPT-3.5] and Microsoft Bing Copilot) were used for translating from English to Polish. Second, 2 native Polish speakers focused on content understanding independently reviewed and corrected the AI-generated Polish version and agreed on a new version. Third, the AI-generated Polish translation was back-translated from Polish into English using the same AI tools. Any discrepancies were discussed by an expert panel consisting of native speakers of English and Polish. This procedure ensured linguistic accuracy and conceptual similarity. Finally, 3 individual cognitive interviews were conducted with native Polish-speaking men to identify whether the questionnaires measured the intended constructs and to find any issues that the respondents might encounter during the response process.

**Results:**

Minor discrepancies between the two AI-generated Polish phrases “umiera z innej przyczyny” and “umiera z powodu innych przyczyn” were merged by native Polish speakers in the PROCASE Knowledge Index. The original questionnaires and the AI-generated questionnaires had minor differences, but they did not affect the meaning of the questions or what was being asked. We conducted individual cognitive interviews (n=3) with participants aged 47 to 74 years. After the interviews, the questionnaires were adjusted with a few changes to make them easier to understand. In the Attitude Scale, the AI-generated Polish translation was changed from “nieco” to “trochę” to align with everyday language and improve understanding.

**Conclusions:**

AI can be an effective tool in the translation process, offering time and resource savings while maintaining accuracy. However, human involvement is still needed to optimize translation.

## Introduction

Translation of questionnaires in research is essential to ensure cultural relevance by making questions understandable and clear for respondents from diverse backgrounds, leading to more accurate data [1]. Translation also increases the reach of the research, allowing for the inclusion of participants who speak different languages, thereby improving the generalizability of the findings. Furthermore, accurate translation maintains the validity and reliability of the questionnaire, ensuring that the translated version measures the same constructs as the original [2]. In international or cross-cultural studies, translated questionnaires allow for comparisons between different groups, facilitating a better understanding of global differences. As international research relies on standardized questionnaires across multiple countries and languages, the need for translation methods that are both methodologically strong and practically feasible has become more apparent. Therefore, the World Health Organization (WHO) introduced the forward-backward translation method in 2007 to ensure this need [3]. This method involves translating the questionnaire from the original source language to the target language (forward translation) and then translating it back to the source language again (backward translation) by a different translator. The original and back-translated versions are then compared with identify differences and ensure linguistic accuracy and conceptual similarity, and the process concludes with cognitive interviews with the target population. This is quite time-consuming, costly, and requires many researchers and experts in the two languages to translate the questionnaires [3]. These practical challenges may limit the feasibility of applying the full translation framework in large, multicountry studies with restricted time and resources.

With the development of artificial intelligence (AI), the use of AI in research has led to a significant change in how scientific research is conducted and data are analyzed [4]. AI offers models that can process large amounts of data with speed and precision [5]. This capability is particularly valued in fields such as medicine, where AI can enhance diagnostic speed and accuracy, optimize treatment plans, and simplify clinical trials [6]. More recently, AI has also been increasingly explored as a tool to support linguistic tasks, including translation, in health and social science research [7]. However, AI may also introduce new errors and hamper transparency due to a lack of clarity regarding the data on which AI is trained. AI translation may struggle with cultural nuances or specific terminology, potentially leading to misinterpretation or lack of clarity in responses [8]. Additionally, some AI systems might not handle complex linguistic structures well, potentially leading to errors in interpreting nuanced survey questions. For questionnaire translations, where accuracy of phrasing can influence survey responses, these issues can be critical [9]. Despite these concerns, AI translation tools are widely used in practice, often without systematic evaluation of how they perform within established translation frameworks. By acknowledging these potential limitations, there may be opportunities to optimize the forward-backward translation process. AI can assist in the translation of the questionnaires and potentially reduce human error and save resources such as time and staffing [10]. Optimizing the translation process through AI may help maintain the high standards of translation accuracy required in cross-country research while improving efficiency. However, evidence remains limited on how AI can be responsibly integrated into standardized translation procedures while preserving linguistic and conceptual accuracy. We aimed to translate questionnaires from English to Polish by incorporating AI into the WHO’s translation process, accounting for potential AI errors by continuing to involve human expertise.

## Methods

### Setting

Prostate cancer (PCa) is the most frequent cancer among men in Europe, with around 450,000 European men diagnosed with PCa each year at an average age of 69 years at the time of diagnosis [11]. Furthermore, approximately 107,000 European men die from PCa per year. Organized screening, including prostate-specific antigen (PSA) testing, can lead to early detection and reduce morbidity and mortality from PCa [12,13]. Today, PCa screening often occurs in an opportunistic setting, which has proven to be ineffective, with no mortality reduction but considerable overdiagnosis, psychological harms, and associated costs [14,15].

This study is part of a 3-year European Union project, PRAISE-U (grant 101101217), in which a systematic multistep PCa screening strategy will be developed, implemented, and evaluated in 4 countries—Poland, Lithuania, Ireland, and Spain (Manresa and Galicia)—including men aged 50 to 69 years. Beyond the clinical measures, the focus is on the evaluation of men's knowledge about and attitudes toward PCa screening, individual risk perceptions, and health literacy. These variables are measured through questionnaires distributed to the men who are invited to PCa screening, applying the Prostate Cancer Screening Education (PROCASE) Knowledge Index [16], Attitude Scale [17], Risk Perception [18], and the Brief Health Literacy Scale for Adults (B-HLA) [19]. Given that this is an international pilot study, it is important to ensure the accuracy and consistency of these questionnaire translations between countries [20].

### Translation Procedure

In this study, we followed the WHO forward-backward translation method, which is widely used to ensure linguistic and conceptual accuracy across languages. The WHO’s forward-backward method involves the following steps:

1. Translating the questionnaire from the original source language to the target language (forward translation) by 2 independent people whose native language is the target language and who are fluent in the original source language.
2. Translating it back to the source language by a different translator (backward translation) by 2 independent people whose native language is the source language and who are fluent in the target language.
3. Comparing the original and back-translated versions.
4. Conducting cognitive interviews with the target population.

**Figure 1:**
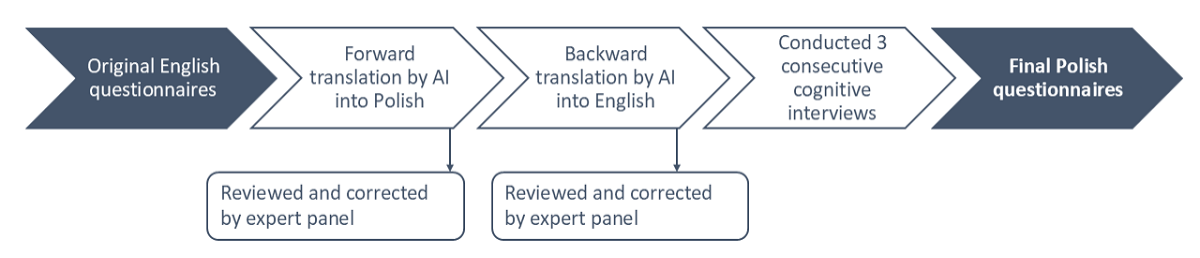
Flowchart of the translation process. AI: artificial intelligence.

The translation process in this study followed an adapted version of the WHO forward-backward method, integrating AI tools to ensure linguistic accuracy and conceptual similarity. The translation process is shown in Figure 1.

Step 1 involved translating the original English versions of the questionnaires into Polish using 2 AI tools: ChatGPT (GPT-3.5; OpenAI) and Microsoft Bing Copilot. These tools were used because they are widely accessible and because of their expertise in language processing and translation tasks. While both models are based on large language models, ChatGPT works as a standalone language model that generates translations based on its internal training data, whereas Microsoft Bing Copilot may draw on web-based information when producing outputs. A simple prompt (“translate to Polish”) was used for both AI tools to evaluate baseline translation performance under realistic conditions, reflecting typical use in which people may not use advanced or detailed prompts. This helped us understand how well their basic translations are and identify areas where more specific instructions could improve the results.

The focus was on assessing whether AI-generated translations are a feasible and useful approach within an established translation framework. Following the AI-generated translations, an expert panel consisting of 2 native Polish speakers was recruited to review and refine the translated Polish versions, with a focus on ensuring linguistic accuracy and conceptual similarity. In this step, the two AI-generated translations were reviewed individually, after which each reviewer independently merged the AI-generated versions into a single document. This process was guided by personal linguistic preferences in Polish and informed by an understanding of the target population for whom the questionnaires were intended. After individual reviews, the two native Polish speakers met to discuss any differences found in the AI-generated translations and agreed on a revised Polish version. This step aimed to address any disparities that might arise during the AI translation process, ensuring the final version was understandable.

To evaluate the performance of the two AI models used for translation (ChatGPT and Microsoft Bing Copilot), we used a structured evaluation framework focusing on translation quality. The evaluation was integrated into the existing WHO forward-backward translation workflow.

Each AI-generated translation was reviewed according to three factors often used in translation research: (1) linguistic accuracy, defined as change of grammar, vocabulary, and syntax; (2) conceptual alignment, defined as maintenance of the original meaning; and (3) clarity and readability, defined as clarity for the target population.

For each questionnaire item, the AI-generated translations were independently reviewed by 2 native Polish language experts during the expert review phase. Revisions were classified using a scoring system:

- No issue—no revision needed.
- Minor issue—wording revision without a change in meaning.
- Major issue—conceptual revision needed.

Scores were noted for translations generated by ChatGPT and Microsoft Bing Copilot. The number and type of issues identified for each AI model were summarized descriptively to allow for a comparison of translation performance. Discrepancies were resolved through expert consensus, resulting in a final revised Polish version.

For step 2, the revised Polish version underwent back-translation into English using the same AI tools used in the initial translation phase (ChatGPT and Microsoft Bing Copilot). This step aimed to assess the consistency and quality of the translated content by comparing it with the original English questionnaire. The 2 AI back-translated versions were merged into a single translated version. This was done by a project manager familiar with the original English questionnaires, and this process was also guided by linguistic preferences in English and an understanding of the target population.

Step 3 consisted of validating the accuracy and conceptual similarity of the translated questionnaire; therefore, an expert panel was convened, consisting of 2 Polish researchers proficient in English and experts in PCa screening, as well as 2 researchers who were either native English speakers or proficient in English and experts in the subject addressed by the questionnaires. The expert panel systematically reviewed the AI back-translated English version against the original English version and provided comments, discussing any discrepancies observed and additionally resolving any linguistic or conceptual challenges. The same project manager involved in Step 2 then merged and revised the AI back-translated English version based on the expert panel’s feedback and consensus.

On the basis of the feedback and comments from the expert panel, a final version of the AI-generated translated questionnaires was refined, ensuring linguistic accuracy and alignment with the original English questionnaires. The finalized Polish version of the questionnaires represented the final step of the forward-backward translation process, validated through collaborative efforts between AI tools and different researchers’ expertise.

### Cognitive Interviews

The fourth and final step involved cognitive interviews. Cognitive interviewing is a qualitative method, and the purpose of this study was to evaluate the quality of the AI-generated Polish-translated questionnaires. This method aims to identify whether the questionnaires measured the intended constructs and to find any issues that the respondents may encounter during the response process [21].

The cognitive interviews were conducted using a structured interview guide to ensure consistency in data collection. Each participant was informed of the purpose of the interview before the start and signed a consent form. Participants were asked to complete the questionnaires one by one. After completing each questionnaire, participants were asked specific questions related to their understanding of the items. Additionally, the interviewer also asked follow-up questions to probe the participant’s initial thoughts and to identify any uncertainties or confusion. Examples of these questions included the following:

What was your initial thought when answering the questionnaire?

Were there any questions you found ambiguous or confusing?

What do you think about when answering these questions?

What considerations do you make before selecting your answer from the options?

In addition to the specific follow-up questions, participants were asked general questions after they had answered all the questionnaires. Examples of these questions included the following:

Is there anything else you would like to add or comment on regarding the questionnaires?

Is there anything else we should know about your thoughts on these questionnaires?

The interviews were recorded to ensure accurate capture of the participants’ responses. Additionally, the interviewer took notes during and after each interview to document any nonverbal signals and to capture initial impressions and observations.

### Ethical Considerations

According to the Consolidation Act on Research Ethics Review of Health Research Projects, Consolidation Act number 1083 of September 15, 2017, section 14(2), health science survey studies and interview-based investigations that do not involve human biological material are exempt from notification to the research ethics committee system. The project was listed in the record of processing activities for research projects in the Central Denmark Region (1-16-02-286-24) according to the European Union’s General Data Protection Regulation. All participants were anonymized, and no identifying information was reported in accordance with standard ethical and reporting practices. Participants did not receive any financial compensation or incentives for participation.

## Results

### AI Translation

#### Forward Translation

For the translation process, we used the same prompt for both AI models and prompted them with “Translate to Polish.”

Similar words and synonyms are terms with identical or closely related meanings. Across the 4 questionnaires (26 items), both AI tools generated translations that were largely comparable to the original English versions. For ChatGPT, 18 of 26 (69.2%) items showed no identified issues, while 6 (23.1%) items were classified as having minor issues and 2 (7.7%) items as having major issues. Similarly, Microsoft Bing Copilot generated 19 (73.1%) translations with no identified issues, 4 (15.4%) with minor issues, and 3 (11.5%) with major issues.

Overall, most identified issues were minor and primarily related to wording, stylistic preferences, or readability rather than changes in meaning.

ChatGPT needed fewer major conceptual revisions compared with Microsoft Bing Copilot, whereas Microsoft Bing Copilot showed fewer minor issues overall. The main problems were cases where words such as “not” were added or left out, which changed the meaning of factual statements, especially in the PROCASE Knowledge Index. All major issues were resolved during the expert review phase, resulting in a final translation that preserved conceptual alignment across all items. Detailed item-level comparisons are available in Multimedia Appendix 1.

To outline some of the minor issues identified, examples from the PROCASE Knowledge Index and Attitude Scale are explained below. For instance, in the PROCASE Knowledge Index, the similarity in meaning between the phrases “...z innej przyczyny” (“...from other causes”) and “z powodu innych przyczyn” (“...due to other causes”) was discussed during the expert review. As both phrasings have the same meaning, the ChatGPT version was selected to keep the sentence shorter without losing its meaning (Textbox 1).

**Example of minor wording differences identified during forward translation of the Prostate Cancer Screening Education Knowledge Index (differences shown in italics).**

- ChatGPT (question 1): Większość mężczyzn zdiagnozowanych z rakiem prostaty umiera z
  innej przyczyny.
- Microsoft Bing Copilot (question 1): Większość mężczyzn zdiagnozowanych z rakiem prostaty umiera z
  powodu innych przyczyn.
- Revision after expert review (merged version): Większość mężczyzn zdiagnozowanych z rakiem prostaty umiera z
  innej przyczyny.

Similarly, in the Attitude Scale, differences were identified in the wording of the response options that were closely related in meaning. For example, the terms “złe rzeczy” (“bad things”) and “niekorzystne” (“unfavorable”), as well as “niezbyt przyjemne” (“not very pleasant”) and “nieprzyjemne” (“unpleasant”) are all similar or closely related words with the same meaning rather than differences in conceptual meaning. The differences between “niezbyt przyjemne” and “nieprzyjemne” are mainly related to the degrees of unpleasantness (Textbox 2).

**Example of minor wording differences identified during forward translation (Attitude Scale).**

ChatGPT (answer options)

- (c) 1: Złe rzeczy, 7: Dobra rzecz
- (d) 1: Przyjemne, 7: Niezbyt przyjemne

Microsoft Bing Copilot (answer options)

-
  (c) 1: Niekorzystne, 7: Korzystne
  (d) 1: Przyjemne, 7: Nieprzyjemne

Revision and correction by native Polish speaker

-
  (c) 1: Niekorzystne, 7: Korzystne
  (d) 1: Przyjemne, 7: Nieprzyjemne

#### Backward Translation

After the 2 AI-translated questionnaires were merged into one by the native Polish experts, this version was translated back into English using the prompt “translate to English” for both AI models. The original questionnaire and the AI-generated translated questionnaire were compared by an expert panel consisting of native English and Polish-speaking researchers. The differences between them are presented in Textbox 3.

**Example of minor wording differences identified during backward translation.**

Prostate Cancer Screening Education (PROCASE) Knowledge Index

- Artificial intelligence (AI) generated (question 1): Most men diagnosed with prostate cancer die
  from other causes.
- Original questionnaire (question 1): Most men diagnosed as having prostate cancer die
  of something else.

Attitude Scale: AI-generated answer option

-
  (c) 1: Unfavorable, 7: Beneficial

Attitude Scale: original questionnaire answer option

-
  (c) 1: Bad thing, 7: Good thing

Overall, only minor differences were observed between the translations generated by ChatGPT and Microsoft Bing Copilot, primarily related to wording and sentence structure rather than conceptual meaning. These differences were resolved during the expert review process and did not affect the final translated versions.

### Cognitive Interviews

The cognitive interviews included men aged 47 to 74 years with no history of PCa. The men were from Poland and had Polish as their native language but resided in Denmark. They were recruited through postings on the hospital’s website or through networks. The men’s professions included a handyman, a researcher, and a retired businessman. The age range was chosen based on the age range selected in the PRAISE-U PCa screening program. However, due to difficulties in finding men aged 50 to 69 years, this range was extended by a few years. One interview was conducted with a Polish translator by profession, one was conducted in English, and one was conducted in Danish.

The cognitive interviews led to some changes in the Polish questionnaire. These changes were made to improve clarity and accuracy based on the feedback from the interviewees. Below are the specific changes described, and Table 1 gives an overview of the changes.

**Table 1:** Overview of the changes after cognitive interviews (change shown in italics).

Interviewee	Questionnaire	Item	AI^a^-generated Polish-translated questionnaire	Change after cognitive interview
2	PROCASE^b^ Knowledge Index	Questions 6-8	PSA^c^	PSA *badanie* *krwi*
3	Attitude Scale	Introduction text	Jeśli Pan uważa, że jest to *nieco* korzystne, proszę zaznaczyć 3	Jeśli Pan uważa, że jest to *trochę* korzystne, proszę zaznaczyć 3
2	B-HLA^d^	Question 1	Mam *adekwatne* informacje na temat zdrowia	Mam *dobre* informacje na temat zdrowia
3	B-HLA	Question 5	Potrafię łatwo podać przykłady *promowania* zdrowia	Potrafię łatwo podać przykłady *poprawy* zdrowia
3	B-HLA	Answer option	Całkowicie *prawda*	Całkowicie *prawdziwe*

^a^AI: artificial intelligence.

^b^PROCASE: Prostate Cancer Screening Education.

^c^PSA: prostate-specific antigen.

^d^B-HLA: Brief Health Literacy Scale for Adults.

In the PROCASE Knowledge Index, the Polish AI-generated translation of “(PSA)” was revised to “(PSA badanie krwi).” The term “PSA” alone was found insufficiently clear for respondents. Adding “badanie krwi” (blood test) provided necessary context, ensuring that participants understand the question refers to the PSA blood test and helps those men who do not know what PSA is.

In the Attitude Scale, the Polish AI-generated translation was “Jeśli Pan uważa, że jest to *nieco* korzystne, proszę zaznaczyć 3” This was revised to “Jeśli Pan uważa, że jest to *trochę* korzystne, proszę zaznaczyć 3.” The term “nieco” (“somewhat”) was changed to “trochę” (“a little”) to better reflect the intended meaning and because this word is more of an everyday term, making the sentence more understandable.

For the B-HLA questionnaire, several changes were made. For question 1, the original translation, “Mam *adekwatne* informacje na temat zdrowia,” was revised to “Mam *dobre* informacje na temat zdrowia.” The word “adekwatne” (“adequate”) was changed to “dobre” (“good”) because it is a more commonly used term and makes the sentence easier to understand.

For question 5, the original translation, “Potrafię łatwo podać przykłady *promowania* zdrowia” was revised to “Potrafię łatwo podać przykłady *poprawy* zdrowia.” The term “promowania zdrowia” (“promoting health”) was revised to “poprawy zdrowia” (“improving health”) to better align with the intended context of providing examples of health improvement.

Last changes in the B-HLA questionnaire were made to the answer options. The original translation, “Całkowicie prawda,” was changed to “Całkowicie prawdziwe.” The term “prawda” (“true”) was changed to “prawdziwe” (“truthful”) to enhance grammar and clarity in the context of a response option and to ensure consistency across response options. Additionally, the word is a more commonly used term.

The cognitive interviews highlighted specific areas where the AI-generated Polish translations could be improved for better understanding. The changes made were essential to ensure that the questionnaire correctly reflects the intended meanings and is easily understood by the respondents.

## Discussion

### Principal Findings

This study explored how AI could help translate questionnaires for a PCa screening study, with the purpose of making the process faster and more efficient. By using AI models for translation, the study found that AI could create Polish translations that were very similar in meaning to the original English versions. While the AI-generated translations were accurate, some minor adjustments were still needed to ensure that the meaning was clear and culturally appropriate. Rather than simply showing that AI can be used to translate questionnaires, this study also evaluated how well AI performs when integrated into an established WHO translation process. By reviewing and comparing AI-generated translations, we provided insight into the strengths and limitations of using AI as part of questionnaire translation in cross-cultural health research.

The original forward-backward translation method is a widely used approach for adapting questionnaires across languages and cultures. The quality of the translation depends heavily on the skills and expertise of the translators involved [22]. Ideally, translators should be fluent in both the source and target languages and have knowledge of the subject matter of the questionnaire. However, even with skilled translators, bias in understanding can influence the translation process. Differences in translators’ understanding, level of education, or perspectives may introduce variations between the original and translated versions. Therefore, validation of the translated questionnaire is an important step to ensure its reliability. Validation involves measuring the psychometric properties of the instrument, such as reliability (internal consistency) and construct validity [23,24]. This process helps confirm that the translated questionnaire measures the same constructs as the original version and is consistent across different populations. Psychometric validation was not done as part of this methodological study, as the primary aim was to assess the feasibility of integrating AI-assisted translation within the WHO workflow. Future research will include reliability testing, construct validity assessment, and cross-cultural equivalence analyses to ensure that the translated instruments measure the same constructs as the original versions.

A strength of this study was the process used to ensure the translations were accurate. When translating questionnaires, it is crucial to have collaboration among stakeholders and involve skilled translators, particularly those with expertise in the subject area [25]. In this study, we used AI as the translator and involved skilled translators and researchers who were native Polish or English speakers to review and correct the AI-generated translations, and expert panels were involved to confirm that the translated questions retained their original meaning. Additionally, it is essential to address cultural sensitivity when translating questionnaires. This involves adapting questions to align with cultural norms and expectations while preserving their original intent [25]. ChatGPT can perform multilingual translations, but it struggles with low-resource languages or dialects not represented in the training data [26]. These challenges include terminology inconsistencies across languages and translation of context-specific phrases, potentially resulting in misinterpretations or unclear responses. However, despite these challenges, AI can improve cross-linguistic communication. Therefore, translation remains dependent on human expertise, because while AI models assist in translation, they cannot replace certified translators in contexts requiring precision and cultural sensitivity [27]. We tried to avoid this by incorporating an expert panel and cognitive interviews with native Polish speakers. The Polish men helped identify any areas where the wording could be confusing, allowing the researchers to make final adjustments. However, this study also showed that AI is not perfect. While the translations were accurate, there were some differences between the AI version and the original English version. While AI models like ChatGPT can efficiently process and generate translations, particularly for preliminary drafts or repetitive tasks that save time [26], they still require human involvement to ensure the translations have the correct meaning, especially when dealing with more complex or sensitive topics. Additionally, AI-assisted translation is often described as a time-saving approach, but this study did not formally measure or compare the time needed for AI-generated translation and expert review with that of a traditional forward-backward translation process. Therefore, any statements regarding efficiency should be interpreted as contextual rather than empirical.

AI continues to develop and improve constantly. A study found that a comprehensive evaluation of ChatGPT’s performance demonstrated remarkable accuracy, consistency, and the ability to improve over time [28]. Therefore, the translation of questionnaires using AI will improve in the future and become better at adapting the language to the target audience as AI continues to improve. In this study, the prompt was intentionally limited to “translate to Polish or English” to evaluate AI performance. Future research could explore whether domain-specific prompts, including information about the questionnaire’s purpose, key terminology, and intended respondents, improve translation quality and conceptual accuracy. Additionally, future work should incorporate comparisons between AI-generated translations and professional human translations. This kind of comparison could give a clearer understanding of the strengths and weaknesses of different translation approaches, help identify where AI may need additional human help, and contribute to the development of evidence-based guidelines for the effective use of AI in questionnaire translation.

The cognitive interview sample was small (n=3), which reflects that this was a study focusing mainly on feasibility. Additionally, after the third cognitive interview, only a few minor changes to the questionnaires were made, and it was therefore assessed that data saturation had been reached and that additional interviews were not necessary. Furthermore, the 3 men participating in the cognitive interviews were residing in Denmark, which may have influenced their language use and may not fully reflect Polish as spoken in Poland. Nevertheless, we considered it preferable to include these participants rather than skip cognitive interviewing altogether, given the practical challenges of recruiting male participants within the project timeframe. In addition, the translation process relied on 2 AI models, selected based on availability, accessibility, and relevance at the time of the study. While the inclusion of additional AI systems could potentially give different results, it remains unclear whether this would alter the overall findings. Further research comparing multiple AI models is therefore relevant.

Finally, the findings from this methodological study are intended to be interpreted in combination with the broader PRAISE-U project outcomes. The translated questionnaires were used within an ongoing multistep screening program, and results from these instruments will be reported alongside PRAISE-U results, allowing further assessment of their performance in real-world research settings.

Translating questionnaires, particularly for health research, requires careful attention to linguistic, cultural, and methodological factors to ensure accuracy and relevance, and it is important to consider multiple aspects, with or without the use of AI. By addressing these considerations, researchers can ensure that translated questionnaires are both linguistically accurate and culturally relevant, improving the reliability and validity of data collected across diverse populations.

### Conclusions

This study shows that AI can play an important role in translating questionnaires without losing their original meaning. While AI models offer efficiency in generating translations, several factors influenced the translation process. These included the role of the expert panel, input from native Polish speakers, and the need to adapt the questionnaires to the target population, which consisted of Polish men. AI helped make the translation process quicker, as it generated translations that were close to the original meaning. Overall, the findings suggest that AI can be a useful support tool in questionnaire translation when combined with human review and a structured evaluation process.

## Appendix

Multimedia Appendix 1 Overview of item-level translation issues identified using ChatGPT and Microsoft Bing Copilot, highlighting conceptual issues and their resolution following expert review.

## Abbreviations

AI: artificial intelligence
B-HLA: Brief Health Literacy Scale for Adults
PCa: prostate cancer
PROCASE: Prostate Cancer Screening Education
PSA: prostate-specific antigen
WHO: World Health Organization

## Data Availability

The datasets generated or analyzed during this study are available from the corresponding author on reasonable request.

## References

[1] Gjersing L, Caplehorn JR, Clausen T (2010). Cross-cultural adaptation of research instruments: language, setting, time and statistical considerations. BMC Med Res Methodol.

[2] Wild D, Grove A, Martin M, Eremenco S, McElroy S, Verjee-Lorenz A, Erikson P, ISPOR Task Force for Translation and Cultural Adaptation (2005). Principles of good practice for the translation and cultural adaptation process for Patient-Reported Outcomes (PRO) measures: report of the ISPOR task force for translation and cultural adaptation. Value Health.

[3] Beaton DE, Bombardier C, Guillemin F, Ferraz MB (2000). Guidelines for the process of cross-cultural adaptation of self-report measures. Spine (Phila Pa 1976).

[4] Stanfill MH, Marc DT (2019). Health information management: implications of artificial intelligence on healthcare data and information management. Yearb Med Inform.

[5] Ray PP (2023). ChatGPT: a comprehensive review on background, applications, key challenges, bias, ethics, limitations and future scope. Internet Things Cyber-Phys Syst.

[6] Topol EJ (2019). High-performance medicine: the convergence of human and artificial intelligence. Nat Med.

[7] Kunst JR, Bierwiaczonek K (2023). Utilizing AI questionnaire translations in cross-cultural and intercultural research: insights and recommendations. Int J Intercult Relat.

[8] Bandakkanavar R (2023). Pros, cons, benefits, and risks of automatic translation tools. krazyTech.

[9] Heruela C (2025). Translating in the digital age: the pros and cons of AI vs. human translation. Tomedes.

[10] Moneus AM, Sahari Y (2024). Artificial intelligence and human translation: a contrastive study based on legal texts. Heliyon.

[11] Hugosson J, Roobol MJ, Månsson M, Tammela TL, Zappa M, Nelen V, Kwiatkowski M, Lujan M, Carlsson SV, Talala KM, Lilja H, Denis LJ, Recker F, Paez A, Puliti D, Villers A, Rebillard X, Kilpeläinen TP, Stenman UH, Godtman RA, Stinesen Kollberg K, Moss SM, Kujala P, Taari K, Huber A, van der Kwast T, Heijnsdijk EA, Bangma C, De Koning HJ, Schröder FH, Auvinen A, ERSPC investigators (2019). A 16-yr follow-up of the European Randomized Study of Screening for Prostate Cancer. Eur Urol.

[12] Andriole GL, Crawford ED, Grubb 3rd RL, Buys SS, Chia D, Church TR, Fouad MN, Gelmann EP, Kvale PA, Reding DJ, Weissfeld JL, Yokochi LA, O'Brien B, Clapp JD, Rathmell JM, Riley TL, Hayes RB, Kramer BS, Izmirlian G, Miller AB, Pinsky PF, Prorok PC, Gohagan JK, Berg CD, PLCO Project Team (2009). Mortality results from a randomized prostate-cancer screening trial. N Engl J Med.

[13] Schröder FH, Hugosson J, Roobol MJ, Tammela TL, Ciatto S, Nelen V, Kwiatkowski M, Lujan M, Lilja H, Zappa M, Denis LJ, Recker F, Berenguer A, Määttänen L, Bangma CH, Aus G, Villers A, Rebillard X, van der Kwast T, Blijenberg BG, Moss SM, de Koning HJ, Auvinen A, ERSPC Investigators (2009). Screening and prostate-cancer mortality in a randomized European study. N Engl J Med.

[14] Roobol MJ (2018). Screening for prostate cancer: are organized screening programs necessary?. Transl Androl Urol.

[15] Arnsrud Godtman R, Holmberg E, Lilja H, Stranne J, Hugosson J (2015). Opportunistic testing versus organized prostate-specific antigen screening: outcome after 18 years in the Göteborg randomized population-based prostate cancer screening trial. Eur Urol.

[16] Radosevich DM, Partin MR, Nugent S, Nelson D, Flood AB, Holtzman J, Dillon N, Haas M, Wilt TJ (2004). Measuring patient knowledge of the risks and benefits of prostate cancer screening. Patient Educ Couns.

[17] Marteau TM, Dormandy E, Michie S (2001). A measure of informed choice. Health Expect.

[18] Fredsøe J, Kirkegaard P, Edwards A, Vedsted P, Sørensen KD, Bro F (2020). A genetic risk assessment for prostate cancer influences patients' risk perception and use of repeat PSA testing: a cross-sectional study in Danish general practice. BJGP Open.

[19] Rasmussen SE, Aaby A, Søjbjerg A, Mygind A, Maindal HT, Paakkari O, Christensen KS (2023). The Brief Health Literacy Scale for Adults: adaptation and validation of the Health Literacy for School-Aged Children questionnaire. Int J Environ Res Public Health.

[20] Guillemin F, Bombardier C, Beaton D (1993). Cross-cultural adaptation of health-related quality of life measures: literature review and proposed guidelines. J Clin Epidemiol.

[21] Balza JS, Cusatis R, McDonnell SM, Basir MA, Flynn KE (2022). Effective questionnaire design: how to use cognitive interviews to refine questionnaire items. J Neonatal Perinatal Med.

[22] Vujcich D, Roberts M, Gu Z, Kao SC, Lobo R, Mao L, Oudih E, Phoo NN, Wong H, Reid A (2021). Translating best practice into real practice: methods, results and lessons from a project to translate an English sexual health survey into four Asian languages. PLoS One.

[23] Hawkins M, Cheng C, Elsworth GR, Osborne RH (2020). Translation method is validity evidence for construct equivalence: analysis of secondary data routinely collected during translations of the Health Literacy Questionnaire (HLQ). BMC Med Res Methodol.

[24] Alharbi K, Alamri AA, Gassas R (2024). Translation, cultural adaptation, and validation of the Arabic version of the Student Evidence-Based Practice Questionnaire (S-EBPQ)". BMC Med Educ.

[25] Ozolins U, Hale S, Cheng X, Hyatt A, Schofield P (2020). Translation and back-translation methodology in health research - a critique. Expert Rev Pharmacoecon Outcomes Res.

[26] Luo X, Deng Z, Yang B, Luo MY (2024). Pre-trained language models in medicine: a survey. Artif Intell Med.

[27] Greńczuk A, Chomiak-Orsa I, Tryczyńska K (2024). AI-supported translation tools for legal texts: a comparative analysis. Procedia Comput Sci.

[28] Gurbuz T, Gokmen O, Devranoglu B, Yurci A, Madenli AA (2024). Artificial intelligence in reproductive endocrinology: an in-depth longitudinal analysis of ChatGPTv4's month-by-month interpretation and adherence to clinical guidelines for diminished ovarian reserve. Endocrine.

