# Supplementary material for: Using AI in Forward-Backward Translation of Questionnaires for Men Invited to Prostate Cancer Screening: Methodological Study
Source: JMIR Form Res. 2026 Feb 26;10:e81900. doi: 10.2196/81900 (PMC12982947; doi:10.2196/81900)
Supplement: Multimedia Appendix 1 [file formative_v10i1e81900_app1.docx]

# Supplementary Table S1

## PROCASE Knowledge Index

| Item | ChatGPT | Co-pilot | Issue type | Description | Final decision |
| --- | --- | --- | --- | --- | --- |
| Q1 | No issue | Minor | Minor | Slight phrasing difference (“z innej przyczyny” vs. “z powodu innych przyczyn”) | Stylistic alignment |
| Q2 | No issue | Major | Major | Negation changed meaning (comparative mortality vs. heart disease) | Reworded |
| Q3 | No issue | Major | Major | Negation introduced (“nie jest najczęstszą”) | Reworded |
| Q4 | No issue | Minor | Minor | Modal verb difference (“nigdy” vs. “może”) | Harmonized |
| Q5 | No issue | Major | Major | Negation altered factual meaning | Reworded |
| Q6 | Minor | No issue | Minor | “nietypowy” vs. “nieprawidłowy” | Terminology aligned |
| Q7 | Major | No issue | Major | Absolute detection claim not negated | Reworded |
| Q8 | No issue | Minor | Minor | Slight phrasing clarity | Stylistic edit |
| Q9 | No issue | No issue | - | - | Accepted |
| Q10 | No issue | No issue | - | - | Accepted |

## Attitude scale

| Item | ChatGPT | Co-pilot | Issue type | Description | Final decision |
| --- | --- | --- | --- | --- | --- |
| a | No issue | No issue | - | - | Accepted |
| b | No issue | No issue | - | - | Accepted |
| c | Major | Minor | Major | Polarity reversed (“Złe rzeczy” vs. evaluative framing) | Reworded |
| d | Minor | No issue | Minor | “Niezbyt przyjemne” vs. “Nieprzyjemne” | Harmonized |

## Risk perception

| Item | ChatGPT | Co-pilot | Issue type | Description | Final decision |
| --- | --- | --- | --- | --- | --- |
| Q1 | No issue | No issue | - | - | Accepted |
| Q1 | No issue | No issue | - | - | Accepted |

## B-HLA

| Item | ChatGPT | Co-pilot | Issue type | Description | Final decision |
| --- | --- | --- | --- | --- | --- |
| Q1 | No issue | No issue | - | - | Accepted |
| Q2 | Minor | No issue | Minor | Verb form (“przedstawić” vs. “dawać”) | Harmonized |
| Q3 | Minor | No issue | Minor | Stylistic phrasing | Accepted |
| Q4 | No issue | No issue |  | - | Accepted |
| Q5 | Minor | Minor | Minor | Readability and flow | Simplified |
| Q6 | Minor | No issue | Minor | Word order | Accepted |
| Q7 | No issue | No issue | - | - | Accepted |
| Q8 | No issue | No issue | - | - | Accepted |
| Q9 | No issue | No issue | - | - | Accepted |
| Q10 | No issue | No issue | - | - | Accepted |
